# Exploring the barriers and facilities migrants face in accessing COVID-19 vaccines in Malaysia: A qualitative study

**DOI:** 10.1371/journal.pone.0326045

**Published:** 2025-06-10

**Authors:** Tharani Loganathan, Amirah Zafirah Zaini, Hazreen Abdul Majid

**Affiliations:** 1 Centre for Epidemiology and Evidence-Based Practice, Department of Social and Preventive Medicine, Faculty of Medicine, University of Malaya, Kuala Lumpur, Malaysia; 2 School of Health and Rehabilitation Sciences, Health Sciences University, Parkwood Campus, Bournemouth, Dorset, United Kingdom; 3 Centre for Population Health, Department of Social and Preventive Medicine, Faculty of Medicine, University of Malaya, Kuala Lumpur, Malaysia; Caleb University, NIGERIA

## Abstract

**Background:**

Malaysia provided COVID-19 vaccines for all residents, regardless of citizenship status. However, the extent of vaccine accessibility for migrant populations remains unclear, given the complex healthcare barriers they face. This study explored the barriers migrants faced in accessing COVID-19 vaccines and the measures taken to facilitate their access.

**Methods:**

This qualitative study, conducted between April 2022 and February 2023, involved 32 purposively selected key informants from non-governmental organisations (7), international organisations (2), labour unions (2), healthcare providers (11) and migrant communities (10). Data were collected through in-depth, semi-structured, primarily individual interviews, and analysed using NVivo 12 Pro software following a six-phase thematic analysis: data familiarisation, code generation, theme identification, theme review, theme definition, and reporting.

**Results:**

Thematic analysis identified barriers—including legal and administrative challenges, digital exclusion, vaccine hesitancy, and logistical issues—and facilitators, such as mandatory vaccination policies, innovative delivery approaches, communication support, and community engagement. Substantial challenges arose from pervasive distrust in healthcare services, compounded by identity document requirements, roadblocks, and fears of immigration arrests. Digital appointment systems excluded many migrants due to language and literacy barriers, data privacy concerns, and the need for identity documents. While vaccine hesitancy among migrants was generally low, concerns were primarily driven by fears of immigration enforcement rather than vaccine safety. Despite vaccination being voluntary, mandatory requirements for digital vaccination certificates or negative COVID-19 test results strongly incentivised uptake. Employer support, driven by economic interests, played a critical role in promoting compliance with workplace vaccination mandates. Collaboration with non-governmental organisations and community partners proved pivotal, offering tailored health communication and building trust. Flexible vaccine delivery strategies, including a shift from centralised to outreach models near residences and workplaces, enhanced access, particularly for hard-to-reach populations.

**Conclusion:**

Despite numerous challenges, Malaysia’s vaccination efforts were largely successful due to innovative and collaborative delivery and communication strategies. To further improve health equity among marginalised populations, it is essential to enhance culturally sensitive training for frontline workers, strengthen employers’ business ethics, and increase the involvement of trusted stakeholders.

## Introduction

COVID-19 vaccines are a global public good. The World Health Organization (WHO) underscores the need for equitable vaccine distribution, particularly for marginalised groups like migrants [1–3]. Migrant populations, particularly those in irregular situations, are disproportionately impacted by the health and socioeconomic impacts of the COVID-19 pandemic, while having limited access to healthcare services, including vaccination [4–9]. Although many countries included migrants in their national COVID-19 vaccination programmes, inconsistencies in implementation, including the exclusion of undocumented migrants, and context-specific barriers to accessing healthcare, resulted in hard-to-reach populations remaining under immunised [10,11].

Malaysia ranks among the top destination countries for international migrants in Southeast Asia, with approximately 2.7 million documented migrant workers recorded in 2023 [12]. However, the total number of migrant workers, including undocumented migrant workers, is estimated to be as high as 5.5 million [13,14]. Additionally, Malaysia hosts a significant number of refugees and asylum-seekers, with 182,010 registered individuals as of July 2023, primarily Rohingya from Myanmar [15].

Malaysia is not a signatory to the international conventions protecting the rights of migrant workers [16] or recognising the rights and status of refugees and stateless persons [17,18]. Malaysian immigration law does not differentiate between refugees or asylum-seekers and undocumented migrants, resulting in limited entitlements to healthcare, social protection and formal employment, and placing them at risk of immigration arrest and detention [19,20].

Malaysia launched its National COVID-19 Immunisation Programme (NIP) in February 2021, offering voluntary and free COVID-19 vaccines to all residents. The government confirmed the eligibility of all foreign nationals, including undocumented migrants, with assurances that seeking vaccination would not lead to immigration arrest [21]. While the NIP has been widely regarded as successful, with high vaccine uptake among the general population [22], research on migrants’ vaccination experiences remains limited. Existing studies have predominantly focused on citizens [23,24], university students [25], Muslims [26], and the elderly [27], overlooking the unique healthcare access challenges faced by migrant populations [28–31].

Migrant populations often encounter systemic barriers in accessing healthcare, including legal uncertainties, language barriers, financial constraints, and fear of deportation [28–30]. Given these complexities, a qualitative approach was chosen to provide an in-depth understanding of the structural, social, and policy-related factors influencing vaccine access. Qualitative methods allow for the exploration of lived experiences, capturing nuances that may be overlooked in quantitative surveys [32]. This study addresses a knowledge gap by exploring the barriers and facilitators migrants faced in accessing COVID-19 vaccines in Malaysia.

### Overview of COVID-19 immunisation programme in Malaysia

Malaysia launched its National COVID-19 Immunisation Programme (NIP) in February 2021, aiming to vaccinate at least 80% of the population, including undocumented migrants, by February 2022. The programme offered free COVID-19 vaccines to all residents, regardless of citizenship or legal status, with assurances that seeking vaccination would not lead to immigration arrests [21].

Two committees, the Special Committee for Ensuring Access to COVID-19 Vaccine Supply (JKJAV) and the COVID-19 Immunisation Task Force (CITF), managed vaccine supply and distribution [33,34]. The NIP initially prioritised frontline workers and high-risk groups, later extending to adults, teenagers, and children, with booster doses introduced in October 2021 [21].

Vaccine appointments were primarily made through the MySejahtera app or the government website (http://www.vaksincovid.gov.my), with manual and walk-in registrations introduced later to support hard-to-reach populations. Vaccinated individuals received digital certificates via MySejahtera and physical vaccination cards [21].

Despite efforts to include non-citizens, challenges persisted. Documentation requirements, fear of immigration arrests, and distrust in authorities deterred many migrants from registering for vaccination [35,36]. Undocumented migrants often lack valid identification and were reluctant to share personal information due to fears of future immigration arrest [31]. Some resorted to using false documents, complicating identity verification at vaccination centres [37,38].

Vaccine administration occurred primarily at designated vaccination centres (PPVs) such as stadiums, convention centres, public halls, and universities. The Bukit Jalil National Stadium mega PPV, capable of administering up to 10,000 doses per day, became a key walk-in vaccination venue for non-citizens [39]. However, issues such as overcrowding and long waiting times reduced efficiency [40].

To enhance vaccine access for migrants, the Ministry of Health (MOH) engaged in a public-private partnership with ProtectHealth Corporation Sdn. Bhd., expanding vaccination through private clinics, workplace programs, mobile clinics, and outreach initiatives [41,42]. More than 2,600 private healthcare providers, including general practitioners, private hospitals, and NGOs, participated in these efforts. Outreach programs also focused on other marginalised groups, including indigenous populations, the elderly, and those in assisted living facilities. The Malaysian Red Crescent Society supported these initiatives, offering alternative registration methods via WhatsApp, hotlines, and the ProtectHealth website [42].

The industrial vaccination programme (PIKAS), launched in June 2021 and coordinated by the Ministry of International Trade and Industry (MITI), targeted workers in key industries, with vaccines administered directly at workplaces [42].

**Fig 1:**
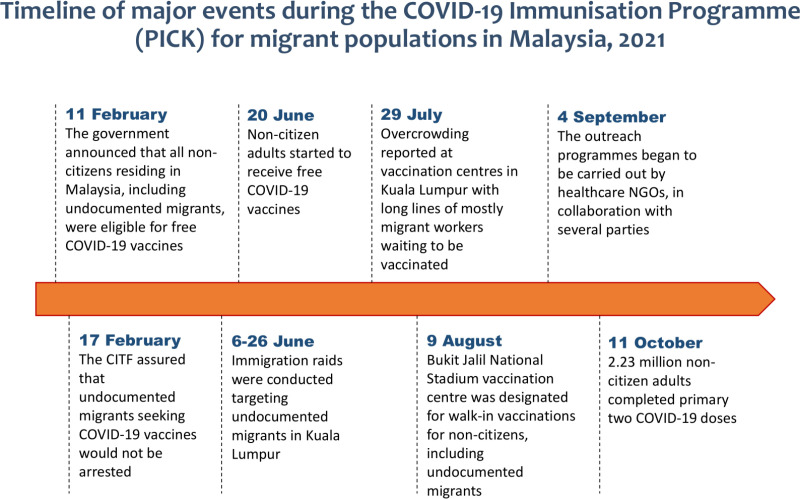
Timeline of major events during the COVID-19 Immunisation Programme (NIP) for migrant populations in Malaysia, 2021.

By 30 May 2023, 84.4% of the country’s population had completed the primary COVID-19 vaccination, including at least 2.23 million non-citizen adults [43,44]. The timeline of major events in Malaysia’s vaccination programme for migrants is presented in Fig 1.

## Materials and methods

### Definition of terms

In this study, the term migrant population refers to documented and undocumented low-income migrant workers, refugees, and asylum-seekers residing in Malaysia. International travellers, students, and expatriates were excluded.

According to the International Labour Organization (ILO), migrant workers are individuals who migrate from one country to another for employment [45]. Documented migrant workers possess the necessary legal documents, such as valid passports and work permits, while undocumented migrant workers either enter a country without the required documentation or overstay their visas, violating immigration terms [46,47].

Refugees are forcibly displaced individuals who cannot return to their country of origin due to a well-founded fear of persecution, as recognised and protected by international law [18]. Asylum-seekers are individuals seeking international protection whose refugee status has not yet been determined [48].

### Sampling and recruitment

We purposively sampled key informants with expertise in migrant health during the COVID-19 pandemic in Malaysia, including representatives from non-governmental organisations (NGOs), international organisations (IOs), labour unions, policy stakeholders, healthcare providers, and migrant communities. Participants were identified during an initial desk review and through the research team’s knowledge of key actors in migrant health. Potential participants were invited to participate in the study via telephone and email, and additional participants were identified through snowball sampling. Recruitment continued until researchers agreed that additional interviews would not yield new information.

### Data collection

Semi-structured interview guides, with questions focused on understanding migrant populations’ access to COVID-19 vaccines in Malaysia, were developed. The interview guides began with introductory questions about the participants’ backgrounds, followed by open-ended questions covering four key topics: (1) vaccine information and acceptance, (2) digital vaccine registration, (3) experience at vaccination centres, and (4) experience with outreach vaccination. The interview guides were tailored to suit two different participant backgrounds: (a) migrant communities, including migrant workers, refugees, and asylum-seekers, and (b) key stakeholders, such as NGOs, IOs, labour unions, policy stakeholders and healthcare workers (public sector, private sector and medical NGOs). See the S1 File for the interview guides.

A total of 29 in-depth interviews with 32 participants were conducted between 6 April 2022 and 20 February 2023, mostly individually, except for three sessions where two participants from the same organisation were interviewed together. The majority of interviews were conducted online, while a few were held in person at either the participants’ offices or our office. The interview mode and location were chosen based on participant convenience to ensure accessibility and safety.

We interviewed 10 participants from migrant communities, including six individuals representing migrant workers from Indonesia, the Philippines, Nepal and Bangladesh, and four individuals representing refugees and asylum-seekers from Myanmar (Rohingya), Iraq and Pakistan. Additionally, we interviewed seven participants from NGOs, two from IOs, and two from labour unions. A total of 11 healthcare providers were also interviewed, including representatives from medical humanitarian organisations, teaching hospitals, primary healthcare services and COVID-19 vaccination centres. Table 1 provides an overview of the study participants.

**Table 1:** Characteristics of the study participants (n = 32).

Participant Background	Label	No.
Migrant Communities		
Migrant Workers	MW	6
Refugees and Asylum-Seekers	REF	4
Non-Governmental Organisations	NGO	7
International Organisations^1^	IO	2
Labour Unions	LU	2
Healthcare Providers		
Medical humanitarian organisations^2^	MHO	4
Teaching hospitals	TH	2
Primary healthcare services	PHS	2
COVID-19 vaccination centres	PPV	3
**Total**		**32**

^1^One of the representatives from IOs interviewed also served as a healthcare provider.

^2^Three of the medical humanitarian organisations were also international organisations.

The interviews were conducted by the research team (AZZ, TL and HM) in either English or Malay, depending on the participants’ preferences. Each interview lasted an average of 50 minutes. Audio recordings were transcribed verbatim, and data analysis was conducted concurrently to refine the interview guides as needed.

### Data analysis

Data analysis was conducted in an immersive, exploratory, and inductive manner, with regular discussions among the research team to refine codes and identify new themes. Thematic analysis was conducted based on Braun and Clarke’s framework [49], which involves six phases: (1) familiarising with the data, (2) generating initial codes, (3) identifying themes, (4) reviewing themes, (5) defining themes, and (6) producing the report.

The research team reviewed the audio recordings and edited the transcripts to ensure accuracy. The transcripts were then coded into emerging themes using NVivo 12 Pro software. To enhance the rigour of the coding process, all transcripts, field notes, and impressions were carefully cross-checked. Regular research team discussions facilitated the refinement of codes, consideration of negative cases, and the identification of minor themes. Interviews conducted in Malay were analysed in their original language, with extracted quotations translated to maintain contextual integrity.

### Validity and reliability

We ensured the rigour of this study through multiple strategies. First, we recruited a diverse range of stakeholders with expertise in migrant health during the COVID-19 pandemic in Malaysia, ensuring a broad representation of perspectives. Recruitment continued until data saturation was reached, enhancing the depth and completeness of the findings. To strengthen validity, we employed triangulation by integrating insights from multiple stakeholder groups and cross-checking findings across different data sources. Additionally, all interviews were conducted using standardised interview guides to ensure consistency, and themes identified during analysis were reviewed and compared across participants to enhance reliability and confirmability.

### Reflexivity

Interviews were conducted by academic researchers from a reputable public university, who could be considered as trusted authority figures. To counter possible power imbalances, participants chose interview times and locations.

### Ethics

To minimise potential harm, anonymity and confidentiality were assured to all participants. During the recruitment process, participant information sheets were distributed and written informed consent was obtained before the commencement of interviews. Participation in this study was entirely voluntary, and participants were informed that they could refuse to answer questions or terminate interviews at any time. Each participant agreed to be audio recorded and to be quoted anonymously in publications. Audio recordings and electronic transcripts were stored in secure data servers. This study obtained ethical approval from the Universiti Malaya Research Ethics Committee (Approval number: UM.TNC2/UMREC_1755).

## Results

The study findings were organised around key barriers faced by migrant populations: (1) legal and administrative challenges, (2) digital exclusion, (3) vaccine hesitancy, and (4) logistical issues. Additionally, measures taken to facilitate vaccine access were categorised into: (5) mandatory vaccination policies, (6) innovative vaccine delivery approaches, and (7) communication support and community engagement. Table 2 summarises the main study findings.

**Table 2:** Main findings of the study.

Theme	Sub-theme	Description
Barriers	Legal and administrative challenges	Lack of trust towards authorities escalated with immigration arrests and mixed messages. Identity document requirements exclude migrants from vaccination centres. Employers were uncooperative fearing business disruptions by immigration enforcement.
	Digital exclusion	Fear of divulging personal information and government surveillance deterred vaccine registration. The use of third-party identities and inconsistent mobile numbers complicated vaccine registration. Lack of digital devices, internet connectivity, and digital illiteracy prevented vaccine registration.
	Vaccine hesitancy	Security concerns overshadowed vaccine hesitancy. Acceptance varied by communities, education levels, home country situations, and misinformation.
	Logistical issues	Difficulty travelling due to roadblocks and fears of potential arrest. Limited capacity for walk-in vaccinations left migrants without alternative lodging and transportation. Substantial hidden costs, including lost wages and transportation costs.
Facilitators	Mandatory vaccination policies	Vaccine requirements at workplaces and public areas were instrumental in overcoming hesitancy.
	Innovative vaccine delivery approaches	Collaborative efforts with trusted partners attributed to the success of outreach vaccination. Mapping exercises facilitated planning, while community engagement increased participation. Involvement of general practitioners and employers facilitated on-site vaccination at workplaces and private clinics. The creation of a unique ID allowed the registration of undocumented migrants.
	Communication support and community engagement	Targeted health messages were translated and disseminated to migrant communities using multiple approaches. Tackling disinformation by sharing personal testimonies and redirecting towards reliable sources.

### Barriers

#### Legal and administrative challenges.

***Lack of trust towards authorities***. The escalation of immigration raids and arrests during the pandemic eroded trust in health authorities despite MOH’s assurances of protection from arrests. As a result, undocumented migrants became increasingly resistant toward government health initiatives, including COVID-19 vaccination.

Initially, when vaccinations were limited to PPVs, participants reported that only documented individuals came forward, while the undocumented migrants hesitated due to fears of immigration-related arrests.

*“Because those who are undocumented, face the risk of arrest. The Home Ministry actually arrested quite a number of migrants during this time. If they were undocumented, they would be arrested. You expose yourself when you turn up at this place for vaccination and then everything is recorded. People were afraid of detention and arrest. Even though the Health Minister said, ‘We won’t arrest (undocumented migrants coming forward for vaccination*)*,’ the Home Ministry never said that they won’t arrest. So, there was a lot of fear of going to government centres for vaccination.”* NGO-005

Contradictory messaging from the MOH and the Ministry of Home Affairs (MOHA) further deepened confusion —while the MOH encouraged migrants to get vaccinated, the MOHA reinforced fears of surveillance and arrest. Participants highlighted migrants’ safety concerns and distrust of government agencies and health authorities. Furthermore, scapegoating and negative public sentiment towards migrants, possibly fueled by official messaging, increased xenophobia and migrants from getting vaccinated.

***Identity documents requirements***. Individuals were required to present passports or other identity documents (IDs) for verification at vaccination centres. While some form of documentation was necessary for registration, healthcare providers did not verify the validity of these documents or report undocumented migrants. Nevertheless, the presence of police officers at vaccination centres intimidated staff, who feared potential repercussions for their employment. As a result, they strictly followed procedures when handling undocumented individuals.

*“There was a point when we had police officers come to our vaccination centre, which made a lot of staff worry about jeopardising their employment. The vaccination centre was run by our own staff nurses, clerks, and medical attendants. They handled registration and entered details into MySejahtera and MyVas (the vaccine administration system*)*. So, if the authorities decided to check how many foreigners had been vaccinated and they could link those names back to the staff, so it was natural for them to be concerned.”* TH-001

Providers emphasised that identity verification before administering vaccinations was essential for ethical practices, ensuring accurate records and preventing fraud, such as multiple doses or identity misuse. However, NGOs highlighted that rigid identity verification posed a significant barrier for non-citizens, particularly undocumented individuals. Asylum seekers without valid IDs relied on community cards that lacked legal recognition, while refugees, despite holding UNHCR cards, remained insecure due to their unrecognised legal status in Malaysia. Even documented migrant workers faced challenges, as employers often held their passports, and uncertainties about work permit renewals further complicated access during the pandemic.

Despite assurances that all individuals would be accepted, frontline staff frequently turned away non-citizens due to expired or invalid documentation. Interviewees stressed the need for proper training to prevent the exclusion of undocumented individuals, as turning them away represented a missed vaccination opportunity.

***Employers were uncooperative***. Study participants informed that employer cooperation was crucial for vaccination efforts, particularly in sectors with large numbers of undocumented workers. However, some employers hesitated, fearing that immigration enforcement could disrupt business operations.

*“The employer was the most difficult (entity*) *to convince, especially in the plantation sector. They were so afraid that no one would be available to harvest their palm oil fruit and that an immigration raid could disrupt their entire business operation. Even when we offered free vaccinations, they rejected them, prioritising concerns that immigration authorities might shut down their businesses over the well-being of their workers.”* NGO-001

Participants also highlighted that export-oriented companies were more likely to comply with vaccination requirements due to responsible recruitment practices and oversight from international bodies like the United States Customs Department, which influenced their access to global markets.

#### Digital exclusion.

***Fear of divulging personal information***. Although the MySejahtera app allowed migrants to register for COVID-19 vaccinations, many were reluctant due to concerns about data privacy and potential government surveillance. Participants noted that migrants, fearing repercussions due to their legal status, hesitated to use the app.

*“Refugees could register on MySejahtera, but many people were still afraid. They wondered, `What does this app mean? Is the Malaysian government tracking me? Does this mean they have all my information and can follow me wherever I go?’ The fear of being undocumented persisted—even for those with UNHCR cards—There was still a lot of fear, and I know many refugees who did not download the app because of it.”* NGO-003

The role of NGOs and community leaders in providing reassurance to migrants was critical in increasing their willingness to receive vaccinations. However, several NGOs interviewed expressed reservations about sharing migrants’ details through the MySejahtera app, citing a lack of trust in government authorities.

***The use of third-party identities and changing phone numbers***. Fearing surveillance through the MySejahtera app, many migrants used false or third-party identities to register for vaccination. In some cases, migrants’ IDs were stolen and used by others, causing discrepancies in vaccination data. The use of false identities complicated healthcare providers’ management of vaccination records, making it challenging to trace vaccination history.

*“We had SO MANY irregular migrants using third-party identities to register on MySejahtera for vaccination. This created another challenge during vaccination. They would show us a card, but the name on it belonged to someone else, and sometimes that person had already left the country.”* NGO-001

Additionally, the MySejahtera system linked individuals’ identification numbers to their phone numbers. Healthcare providers explained that this was problematic, as migrants frequently changed mobile SIM cards, and thus phone numbers. This made it challenging to track vaccination records, jeopardising the administration of subsequent vaccine doses and the generation of digital vaccination certificates.

Healthcare providers often faced challenges with registration errors, missing records, and other issues, requiring extensive collaboration with the MySejahtera helpdesk for resolution. Language barriers further complicated communication, making it difficult to relay complex instructions to migrants and exacerbating problems during the registration process at vaccination centres.

***Lack of digital devices, internet connectivity, and digital illiteracy***. The reliance on the MySejahtera app was problematic, particularly due to the need for smartphones and internet connectivity. Refugees often shared a single smartphone among family members, and poor internet access in rural areas further complicated registration.

Participants noted that the app posed significant challenges for migrants with limited understanding of Malay or English. Language barriers were exacerbated by digital illiteracy, particularly among individuals with lower literacy levels, making it difficult for them to navigate the app for vaccination registration and health information.

*“As I mentioned, my people (the Rohingyas*) *are illiterate, so I have to help them to install the apps. I need to assist everyone and teach them how to use the app. It was a very long process—everyone came to me for help with the MySejahtera app. Digital literacy is the problem. They don’t even know how to use mobile phones”* REF-001

#### Vaccine hesitancy.

***Security concerns overshadowed vaccine hesitancy***. Vaccine hesitancy among migrants appeared to be relatively low, with concerns primarily centred around immigration enforcement rather than on vaccine safety. Study participants agreed that migrants were more willing to be vaccinated compared to Malaysians, largely due to vaccine mandates at workplaces and public venues such as malls and clinics.

The decision to vaccinate was primarily driven by job requirements and livelihood concerns, while worries about adverse events following immunisation were less common among migrants. Migrant workers, in particular, had shown greater willingness to comply with vaccination efforts, attending vaccination appointments in large numbers due to workplace vaccination requirements. Healthcare providers noted that, unlike Malaysians, non-citizens typically did not question vaccine safety or origin, but instead readily accepted vaccination.

*“The citizens are the ones who have a lot of hesitancy. Foreigners are more than willing to come, even in the early phase of PIKAS, especially when they were brought in by the bus loads. I would say this is largely due to policy—if workers don’t get vaccinated, they could be deported, fined, or face other consequences. For foreigners, as long as the government implements the policy, they have to comply, whether they like it or not. From my experience on the ground, I don’t see much questioning from migrants, unlike Malaysians who ask, ‘Is this safe? Is it expired? Where is it from? What brand is it?’ Migrants simply come, get vaccinated, and leave.”* PPV-003

***Acceptance varied by communities***. Participants reported varying levels of willingness to receive the COVID-19 vaccines among migrant communities. This variation was influenced by factors such as education, livelihood considerations, the situation in their home countries, and the spread of misinformation, often through social media networks, including those originating from their home countries.

Community leaders noted isolated cases of hesitancy, often driven by family influence or rumours circulating on social media platforms. These rumours, particularly about adverse health effects, caused concern, especially among individuals with pre-existing health conditions, leading to reluctance to vaccinate.

*“Many didn’t take the vaccine because they are afraid. Rumours spread on Facebook, or friends would say that getting vaccinated would make you crazy, cause death, difficulty breathing, or worsen your health <laughs>. And they believe those rumours. People with diabetes or high blood pressure were even more hesitant.”* REF-003 (Translated from Malay)

In refugee communities, resistance to COVID-19 vaccination was generally lower than initially expected. However, Arab-speaking communities, particularly those from war-torn countries like Syria, showed greater resistance due to distrust in the government and familial discouragement, requiring additional persuasion.

#### Logistical issues.

***Travel restrictions and security checks***. The Movement Control Orders (MCOs) restricted individuals’ movements, with roadblocks set up by authorities. Some migrants were assigned vaccine appointments at distant PPVs, including in other states. As a result, migrants faced travel difficulties, even with scheduled appointments, due to fears of roadblocks, the need for approval to cross state borders, and the risk of immigration arrests, particularly for those without documentation. This led to missed appointments, frustration, and logistical challenges, preventing migrants from accessing COVID-19 vaccination services.

*“A lot of people were hesitant to travel due to all those restrictions... Even with the UNHCR card, many people were afraid they might get stuck at a roadblock … Even if they have an appointment for vaccination, there was still the risk of getting arrested... So, there was a lot of fear around these issues.”* NGO-003

***Limited capacity for walk-in vaccination***. While walk-ins were eventually allowed at some PPVs, vaccination opportunities remained limited. The limited daily capacity led to overcrowding and long waiting times, with migrants often waiting five to eight hours for vaccinations. Furthermore, if they were unable to receive vaccinations on the same day, migrants—particularly those without transportation—faced difficulties in finding accommodation. Language barriers persisted at PPVs, with limited translators, prolonged the processing times and contributing to extended waiting periods.

*“One of the common challenges, even for Malaysians, was the distance from their homes to the vaccination centre... For migrants, it was even harder—imagine travelling a long way, and if they were be told that, ‘Oh, we are out of vaccine’. Migrants are pretty much screwed. They’re left stranded with no place to stay, having to wait until the next day. While the intention behind opening certain PPVs for walk-ins was good, the lack of proper information and social protections made it challenging.”* NGO-002

***Substantial hidden costs.*** Participants highlighted the substantial hidden costs faced by migrants despite the free vaccination programme, including income loss and transportation costs. As many migrants relied on daily wages, they risked unpaid absences from work to get vaccinated, with some even experiencing wage deductions to cover transportation costs to vaccination centres. Additionally, private clinics providing vaccinations reportedly charged migrants high fees, despite the availability of free vaccines.

*“There were instances when employers registered whole groups of migrants for vaccination. So, they arranged buses for transport, but employers charged migrants 100 ringgit per person for logistical arrangements. When private clinics began vaccinating migrants, reports emerged of individuals paying as much as 300 to 400 ringgit to receive a full dose of the vaccine.”* NGO-006

### Facilitators

#### Mandatory vaccination policies.

***Vaccine requirements at workplaces and public areas***. Migrants’ initial reluctance toward vaccination stemmed primarily from concerns about data privacy and registration challenges, with misinformation about adverse effects playing a lesser role. Ultimately, the need for vaccination to return to work was a key factor in overcoming hesitancy and ensuring compliance.

*“Those who work, all want to be vaccinated—by hook or by crook—because companies required vaccination to return to work. Those who refused were mostly stateless individuals or those in informal work. For me, almost every migrant wanted to be vaccinated.”* MHO-001

The Malaysian government’s policy of requiring vaccination certificates for entry into various premises, along with restrictions such as mandatory weekly COVID-19 tests for the unvaccinated, served as a strong motivator for uptake. Employers, facing the risk of operational closures and financial penalties, also enforced vaccination by imposing workplace restrictions, prompting many migrants to comply.

Although vaccination remained voluntary in Malaysia, strong government encouragement incentivised employers to facilitate access, often by arranging transportation to PPVs or organising on-site vaccination sessions with medical officers to expedite the process and minimise disruptions.

#### Innovative vaccine delivery approaches.

***Collaborative efforts with trusted partners***. Undocumented migrants faced significant barriers to vaccination due to fears of immigration arrest, despite reassurances from the MOH. NGOs and IOs advocated for outreach vaccination initiatives, recognising undocumented migrants’ apprehension toward authorities. Reports of overcrowding and extortion following the introduction of walk-in vaccinations further raised safety concerns. In response, the government implemented outreach programmes, allowing trusted NGOs and community partners to facilitate migrant access to vaccination.

The success of these efforts relied on collaboration among multiple organisations, including the Malaysian Red Crescent Society (MRCS), local NGOs, and IOs. The MRCS played a critical role in providing free vaccination services, ensuring protection from arrest, and offering logistical support, particularly in remote areas. Community leaders and social mobilisers served as key intermediaries, leveraging established trust within migrant communities. Coordination between these organisations and district health authorities was essential to the effectiveness of vaccination initiatives.

Interviewees emphasized that trust was crucial, as migrants were more receptive when approached by familiar individuals with longstanding relationships.

“*Vaccinating irregular migrants was not an easy task. It was not simply a matter of offering free vaccination and expecting them to come forward. Gaining their trust was essential. If, for example, Dr. X entered the community and said, ‘I’m here to vaccinate you,’ they would all run away because they did not know him. But when I entered, I provided food, helped their children obtain birth certificates, and assisted those wanting to return home with travel documents. It was through YEARS of interaction and trust that I was able to reach them.”* NGO-001

The outreach vaccination programme was further strengthened by clear messaging from government leaders, reinforcing the commitment to vaccine accessibility for all.

***Mapping exercises and community engagement strategies***. Mapping exercises, conducted with the help of community leaders, were essential for identifying migrant populations in need of vaccination, particularly undocumented and hard-to-reach groups. These efforts helped locate areas with high migrant concentrations, guiding the selection of vaccination sites, and ensuring efficient outreach planning and execution.

*“Mapping where these individuals could be found, estimating their numbers to determine the required vaccine vials, and then discreetly obtaining and delivering the vaccines—this was our approach. We conducted extensive mapping and collaborated closely with numerous NGOs and community gatekeepers.”* MHO-001

Mobile clinics were strategically placed near migrant communities, often close to workplaces, residences, or key gathering sites, such as prayer halls for the Rohingya Muslim community. In some cases, mobile teams traveled to remote areas, including jungle interiors, to reach isolated populations. To enhance awareness and participation, initiatives like live Facebook broadcasts and outreach through community leaders were implemented.

Participants confirmed that vaccination data was securely stored, with only minimal details —such as names, genders, and general locations—shared with the MOH for vaccine distribution purposes. Crucially, this information was not disclosed to police or immigration authorities.

***Involvement of general practitioners and industries***. Despite ongoing NGO-led outreach efforts, some migrants remained unaware of vaccination opportunities or continued to face logistical barriers. Involving general practitioners (GPs) from the private sector proved effective, as they were trusted by undocumented migrants and more accessible than public health clinics.

*“Some who can afford it seek vaccination privately. That’s why we initially advocated for involving GPs in the vaccination effort. GPs are present nationwide and are the most trusted by non-citizens, especially the undocumented, who avoid public clinics due to their lack of documentation. Instead, they turn to private doctors, where they can pay for treatment without fear.”* IO-002

To encourage undocumented migrants to get vaccinated, employers played a key role by facilitating worker transport to PPVs. In later stages, GPs administered on-site vaccinations directly at workplaces through the PIKAS programme, which was particularly beneficial for companies facing logistical challenges in sending employees to vaccination centres.

***Creation of a unique ID***. The introduction of the CITF ID system was a key measure in ensuring equitable access to COVID-19 vaccines for undocumented migrants and other non-citizens in Malaysia. By generating unique serial numbers based on personal details such as name, nationality, age, and gender, this system enabled registration at vaccination sites without requiring formal identity documents. This approach addressed the barriers faced by undocumented individuals, allowing them to access vaccination services.

*“After deliberation with the Health Ministry on ensuring vaccine access for undocumented individuals, a unique code was introduced. This code, generated based on birth dates and other details, enabled undocumented migrants to register for vaccination. It proved effective, particularly when walk-in vaccinations were allowed. Previously, MySejahtera required a document number for registration, creating barriers for those without identification. The unique code system helped bridge this gap and improve vaccine accessibility.”* MHO-002

NGOs informed that the MySejahtera app did not strictly enforce identification number formats, allowing various number sequences for registration. However, feedback highlighted issues, such as passport numbers overlap from different countries. The CITF ID system resolved these conflicts by generating unique serial numbers, ensuring smoother registration.

To accommodate those without smartphones or digital literacy, manual appointment cards were also issued. These cards recorded vaccination details and upcoming appointments, ensuring accessibility for all recipients.

#### Communication support and community engagement.

***Targeted messaging and multipronged dissemination strategy***. Efforts to engage non-citizens in vaccination faced significant challenges due to unclear communication. Official health messaging was primarily available in local languages, making it inaccessible to illiterate Rohingya and other language minorities. NGOs, IOs, and community leaders played a crucial role in translating public announcements and disseminating essential vaccination information to diverse communities, ensuring broader accessibility.

To enhance understanding and address fears, official MOH videos were translated into multiple languages, and short audio recordings were created. Social media was instrumental in spreading information on vaccination procedures and dispelling concerns. Additionally, NGOs conducted counselling sessions at mobile clinics and held face-to-face meetings, ensuring that even those with limited digital access received accurate information. These multipronged, community-tailored strategies aimed to maximise engagement and improve vaccination participation.

*“Lack of education is very problematic for migrants and refugees who do not understand English. To address this, we created videos, and community members also made instructional videos on registering for MySejahtera and vaccination. Additionally, we provided helpers—educated individuals who assisted those with low literacy in understanding the process.”* REF-002

***Tackling disinformation through personal testimonies***. Community leaders leveraged personal testimonies of their own COVID-19 vaccination experiences to combat misinformation and normalise vaccination. These firsthand accounts provided tangible evidence to counter prevailing fears and rumours.

*“What we did was simple—I got my first vaccination in July and shared my experience with them. Nothing happened to me after vaccination, which served as proof that the vaccine was safe and that it was better for us to get vaccinated.”* MW-004

In Sabah, East Malaysia, where misinformation was widespread, community health volunteers (CHVs) conducted door-to-door visits to address concerns and promote vaccination. Participants highlighted social media as a key driver of vaccine hesitancy, underscoring the need for targeted health education and efforts to redirect individuals to reliable information sources.

## Discussion

Vaccinating non-citizens in Malaysia presented significant challenges due to widespread distrust in government and health services. This distrust was compounded by identity document requirements, police roadblocks, immigration arrests, and discriminatory discourses that scapegoated non-citizens as disease carriers [50–52]. Although the MOH offered amnesty from arrest, trust was eroded by longstanding policies obligating health professionals to report undocumented migrants seeking care [29,53], along with the escalation of pandemic-related immigration arrests [31,51,52]. This situation mirrors migrants’ distrust of the United Kingdom’s National Health Service (NHS), shaped by pre-pandemic ‘hostile environment’ policies [54], which continued to influence vaccination decisions despite the United Kingdom’s inclusive COVID-19 vaccine rollout [10,55,56].

Relying on digital applications for vaccination appointments likely excluded non-citizens due to language and literacy barriers, as well as concerns over data security and surveillance. Additionally, electronic vaccination systems that required ID numbers for appointment bookings systematically excluded undocumented migrants—a challenge observed not only in Malaysia but also in Europe, the United States, and South Africa [10,57,58].

Although migrants are often perceived as vaccine-hesitant, our findings indicate relatively low vaccine hesitancy among migrants in Malaysia, with concerns centred more on security rather than vaccine safety. The need for vaccination certificates to travel, work and access education and healthcare services was a strong motivator for uptake. When critically examined, the notion of vaccine hesitancy among migrants may uncover structural barriers often mistaken for individual reluctance [57,59,60]. In contrast to the United Kingdom and Canada, where migrants exhibited higher hesitancy than citizens [57,59,60], a systematic review of barriers and facilitators to COVID-19 vaccine access found that 5 out of 6 selected studies reported high vaccine acceptance among migrants in various countries, demonstrating their general willingness to receive COVID-19 vaccines [61]. In Malaysia, survey data showed that 81% of refugee respondents wanted COVID-19 vaccination. Despite this willingness, many expressed concerns about vaccine side effects and the ability to return to work post-vaccination, highlighting the influential role of friends, family and community leaders in their decision-making [62]. Our study supports these findings, showing that migrants’ primary motivation for vaccination was livelihood and security needs rather than safety concerns. These insights highlight the importance of leveraging trusted networks for effective health communication and vaccination outreach.

In Malaysia, innovative strategies such as walk-in clinics and unique ID generation improved vaccine accessibility. Shifting from centralised to outreach vaccination enhanced convenience and safety for hard-to-reach populations through collaborations with trusted partners who acted as cultural mediators and navigators. Building trust through partnerships with NGOs and community organisations was crucial for vaccinating marginalised non-citizens, particularly undocumented migrants, as seen globally [63,64]. In Japan, NGOs established multilingual call centres and consultation counters to provide vaccine information and facilitate appointments [63]. In Thailand, Migrant Health Volunteers (MHVs) delivered key messages in accessible languages, supported migrants in vaccination processes, assisted local health authorities in mapping at-risk communities, and maintained communication channels to build trust and improve vaccine understanding [65,66]. In Sabah, East Malaysia, Advocates for Non-discrimination and Access to Knowledge (ANAK), a grassroots organisation serving stateless, undocumented, and migrant communities, employed community-based social listening approaches, including community journaling and online focus group dialogues, to facilitate bidirectional communication and deliver tailored messaging [67].

Vaccination in Malaysia is voluntary [68], but the public was strongly encouraged to receive primary and booster doses to reduce disease transmission, severity, and mortality. Digital vaccination certificates or negative COVID-19 test results became mandatory for accessing many venues. The escalation of workplace clusters, notably the Teratai cluster [69,70], led to worksite closures and operational halts, serving as a strong incentive for vaccination [71]. Employers, driven by economic considerations, played a critical role in promoting vaccination among migrants, as compliance with these requirements became essential in work settings [72].

This study has several limitations. While the qualitative design allows for in-depth exploration, the findings may not be generalisable beyond the Malaysian context. However, engaging multiple stakeholders enhances the credibility of the findings through triangulation.

Despite these limitations, the study offers significant strengths. It addresses a critical gap in the literature by examining vaccine access among marginalised migrants in Malaysia—a topic with limited academic research and official statistics. The qualitative approach captures diverse perspectives through interviews with migrants, healthcare providers, and key stakeholders, providing valuable insights into the complexities of vaccine uptake.

## Conclusion

Our study highlights the substantial barriers marginalised migrants faced in accessing COVID-19 vaccines, despite the availability of free vaccination in Malaysia. Successful vaccination efforts hinged on innovative delivery and communication strategies, bolstered by strong collaboration with NGOs and community partners. Emphasising trust-building, effective communication, and creative approaches, our findings stress the need for multipronged strategies to advance health equity, particularly for marginalised populations. While Malaysia’s vaccination efforts were largely successful, further enhancing health equity requires culturally sensitive training for frontline workers, strengthened employer ethics, and greater involvement of trusted stakeholders.

## Supporting information

S1 File for interview guides
(PDF)
