# Supplementary material for: Exploring the barriers and facilities migrants face in accessing COVID-19 vaccines in Malaysia: A qualitative study
Source: PLoS One. 2025 Jun 10;20(6):e0326045. doi: 10.1371/journal.pone.0326045 (PMC12151388; doi:10.1371/journal.pone.0326045)
Supplement: S1 File — for interview guides (PDF) [file pone.0326045.s001.pdf]

Interview Guide on COVID-19 Vaccination Programme

A. **<u>Migrants: Migrant Workers, Refugees, and Asylum-seekers</u>**
Interview topics and questions that form the broad framework of discussion with migrants on the the COVID-19 vaccination programme will include:

Topics:

- Knowledge, experience and perception of COVID-19 vaccine information and acceptance.
- Knowledge, experience and perception on the use of the MySejahtera application.
- Knowledge, experience and perception of the COVID-19 vaccination programme at designated centers (PPV) in Malaysia.
- Knowledge, experience and perception of the COVID-19 vaccination outreach programme in Malaysia.
- Knowledge, experience and perception of the COVID-19 booster doses.
- Knowledge, experience and perception of the COVID-19 vaccination programme for kids.
- Suggestion for improvement of health for migrants in Malaysia.

Introductory questions:

i. Sex
ii. Year of Birth
iii. Nationality
iv. Level of education
v. Years, location, living arrangement in Malaysia
vi. Employment or source of income
vii. Documentation status

Open questions:

1. Could you please share how you received information about the COVID-19 vaccination programme in Malaysia?
   <u>Prompts</u>: Dissemination of information efforts (social media, physical outreach, etc)? Understanding? Vaccine hesitancy? Barriers (language etc)? What triggered you to get the COVID-19 vaccine?

2. How was your (or your friends) experience with the MySejahtera application to get the COVID-19 vaccine in Malaysia?
   <u>Prompts</u>: Registration (access to smartphone, local phone number, ID and etc)? Undocumented migrants? Barriers (language, documents, IT, internet connection etc)? Supports? Other medium (Selangkah)?

3. How was your (or your friends) experience in getting vaccinated at designated centers (PPV) in Malaysia?
   <u>Prompts</u>: Appointment? Logistic? Registration? Undocumented migrants? Role of employers? Barriers (language, document etc)? Supports (translation etc)? Discrimination (enforcement authorities etc)? Challenges for you and how you overcome it? Do you think you received an effective vaccine? Digital vaccine certificate?

4. How was your (or your friends) experience with the COVID-19 vaccination outreach programme in Malaysia?
   Prompts: Information (social media, friends etc?) Role of employers? Barriers (language, document etc)? Supports (translation etc)? Challenges for you and how you overcome it? Do you think you received an effective vaccine? Digital vaccine certificate?

5. How was your (or your friends) experience with COVID-19 booster doses in Malaysia?
   Prompts: Dissemination of information efforts (social media, physical outreach, etc)? Booster hesitancy? Appointment? Undocumented migrants? Role of employers? Barriers (logistic, language, document etc)? Supports? Do you think you received an effective vaccine?

6. How was your (or your friends) experience with COVID-19 vaccination programme for kids in Malaysia?
   Prompts: Dissemination of information efforts (social media, physical outreach, etc)? Vaccine hesitancy? Logistic? Outreach programme? Registration? Undocumented migrant children? Role of employers? Barriers? Supports?

7. Do you have any suggestions for the improvement of health for migrants in Malaysia?

B. **<u>Key stakeholders: Healthcare Providers, NGOs, International Organisations, Labour Unions, Policy stakeholders and etc.</u>**
Interview topics and questions that form the broad framework of discussion with key stakeholders involved in the COVID-19 vaccination programme for non-citizens in Malaysia will include:

Topics:

- Knowledge, experience and perception of COVID-19 vaccine information and acceptance among migrants in Malaysia.
- Knowledge, experience and perception on the use of the MySejahtera application among migrants in Malaysia.
- Knowledge, experience and perception of the COVID-19 vaccination programme at designated centers (PPV) among migrants in Malaysia.
- Knowledge, experience and perception of the COVID-19 vaccination outreach programme among migrants in Malaysia.
- Knowledge, experience and perception of the COVID-19 booster doses among migrants in Malaysia.
- Knowledge, experience and perception of the COVID-19 vaccination programme for kids among migrant children in Malaysia.
- Suggestion for improvement of health for migrants in Malaysia.

Introductory questions:

i. What is your or your organization's role during COVID-19 pandemic for migrants? (activities, success, challenges)

Open questions:

1. Could you please share how information related to the COVID-19 vaccination programme reached the migrant populations in Malaysia?
<u>Prompts</u>: Dissemination of information efforts (social media, physical outreach, etc)? Understanding? Vaccine hesitancy? Barriers (language etc)?

2. Could you please share how migrants access the MySejahtera application to get the COVID-19 vaccine in Malaysia?
<u>Prompts</u>: Registration (access to smartphone, local phone number, ID and etc)? Undocumented migrants? Barriers (language, documents, IT, internet connection etc)? Supports? Other medium (Selangkah)?

3. Could you please share the experience of migrants in getting vaccinated at designated centers (PPV) in Malaysia?
<u>Prompts</u>: Appointment? Logistic? Registration? Undocumented migrants? Role of employers? Barriers (language etc)? Supports (translation etc)? Discrimination (enforcement authorities etc)? Challenges for you or your organization and how you overcome it? Digital vaccine certificate?

4. Could you please share how the COVID-19 vaccination outreach programmes are conducted for migrants in Malaysia?
<u>Prompts:</u> Location of migrants? Role of employers? Barriers (logistic, language etc)? Supports? Challenges for you or your organization and how you overcome it? Digital vaccine certificate?

5. Could you please share how the experience of migrants with COVID-19 booster doses in Malaysia?
   <u>Prompts:</u> Dissemination of information efforts (social media, physical outreach, etc)? Booster hesitancy? Access to vaccination appointment? Undocumented migrants? Role of employers? Barriers? Supports?

6. Could you please share how the experience of migrant children with COVID-19 vaccination programme for kids in Malaysia?
   <u>Prompts:</u> Dissemination of information efforts (social media, physical outreach, etc)? Vaccine hesitancy? Access to vaccine designated centers (PPV)? Outreach programmes? Undocumented migrant children? Role of employers? Barriers? Supports?

7. Do you have any suggestions for the improvement of health for migrants in Malaysia?
